# miR-361-3p Regulates Liver Tumor-initiating Cells Expansion and Chemo-resistance

**DOI:** 10.7150/jca.52395

**Published:** 2021-01-01

**Authors:** Shuping Qu, Xiaobing Zhang, Yue Wu, HengYu Li, Jian Zhai, Dong Wu

**Affiliations:** 1 Department of Hepatic Surgery, Third Affiliated Hospital of Second Military Medical University, Shanghai, 200438, China.; 2 Department of General surgery, First Affiliated Hospital of Second Military Medical University, Shanghai, 200433, China.

**Keywords:** Tumor-initiating cells, hepatocellular carcinoma, miR-361-3p, SOX1, cisplatin, sorafenib

## Abstract

Increasing evidence shows that liver tumor-initiating cells (T-ICs) closely associated with the progression, metastasis, recurrence and chemo-resistance of hepatocellular carcinoma (HCC). However, the underlying mechanism for the propagation of liver T-ICs remains unclear. Here we show that miR-361-3p is upregulated in liver T-ICs. Knockdown of miR-361-3p impairs the self-renewal and tumorigenicity liver T-ICs. Conversely, forced miR-361-3p expression enhances the self-renewal and tumorigenicity liver T-ICs. Mechanistically, miR-361-3p directly targets SOX1 via binding its 3'-UTR in liver T-ICs. Moreover, miR-361-3p knockdown hepatoma cells are more sensitive to cisplatin or sorafenib treatment. Clinical cohort analysis demonstrates that miR-361-3p low HCC patients are benefited from TACE (transcatheter arterial chemoembolization) or sorafenib treatment. In conclusion, our findings revealed the crucial role of the miR-361-3p in liver T-IC expansion and TACE or sorafenib response, rendering miR-361-3p an optimal target for the prevention and intervention in HCC.

## Background

With nearly 700,000 new cases per year, hepatocellular carcinoma (HCC) ranks as the sixth most common cancer in the world 1. However, the early symptoms of HCC are not obvious. Most HCC patients are diagnosed at advanced stage 2. Due to high grade invasive HCC can develop to life threatening metastases and less than 20% patients with Portal vein thrombus-only have 2-year overall survival 3. Currently, most of drugs which used for clinical HCC patients were proved to be disappointed. So, the underlying mechanism of HCC initiation and progression needs to be deeply explored.

Tumor-initiating cells (T-ICs) or cancer stem cells (CSCs) are a subgroup of cancer cells, which have the self-renewal ability and tumorigenesis capacity 4, 5. T-ICs or CSCs were first discovered in blood system diseases 6. Later studies also confirmed the existence of T-ICs or CSCs in solid tumors, including breast cancer, liver cancer and glioma 7-9. Currently researches suggest that T-ICs or CSCs are responsible for the progression, metastasis, recurrence and chemo-resistance of cancers 10-12. Therefore, identification of the underlying mechanisms governing T-ICs or CSCs propagation may lead to the discovery of promising therapeutic strategies for cancer patients.

MicroRNAs (miRNAs) are a class of small noncoding RNA molecules that contain approximately 22 nucleotides 13. It was reported to be essential for many physiological processes, such as cellular homeostasis, development, differentiation, cell survival and death, tumor initiation and progression 14, 15. miR-361-3p is a newly discovered miRNA, and its function and mechanism of action in biological processes and diseases are not completely understood. Previous studies found that miR-361-3p promotes human breast cancer cell viability by inhibiting the E2F1/P73 signaling pathway 16. Moreover, miR-361-3p regulates ERK1/2-induced EMT via DUSP2 mRNA degradation in pancreatic ductal adenocarcinoma 17. However, the function of miR-361-3p in liver T-ICs is unclear.

In this study, we for first find that miR-361-3p is highly expressed in liver T-ICs. Next, by using loss-of-function analysis and gain-of-function analysis in liver T-ICs, we demonstrate that miR-361-3p promotes the self-renewal capacity and tumorigenicity of liver T-ICs. Further mechanism study reveals that miR-361-3p directly targets SOX1 in liver T-ICs. Interestingly, miR-361-3p knockdown hepatoma cells are more sensitive to cisplatin or sorafenib treatment. Clinical cohort analysis demonstrates that miR-361-3p low HCC patients are benefited from TACE or sorafenib treatment. Altogether, we discover that miR-361-3p promotes the expansion of liver T-ICs via interacting with SOX1.

## Materials and Methods

### Cell Culture

Huh7 and HepG2 cells were maintained at 37°C in 5% CO_2_ incubator with Dulbecco's modified Eagle's medium (DMEM) supplemented with 10% fetal bovine serum (FBS), 2 mM L-glutamine, and 25 µg/ml of gentamicin. FBS was purchased from Nova-Tech (Grand Island, NE). miR-361-3p mimic virus or miR-361-3p sponge virus was purchased from RIBOBIO (Guangzhou, China). siRNA SOX1 was purchased from Genechem (Shanghai, China).

### Patients and specimens

HCC tissues were obtained from patients who underwent liver resection in the Eastern Hepatobiliary Surgery Hospital (EHBH). Samples were frozen in liquid nitrogen immediately after surgical resection for further RNA extraction. A total of 70 patients received TACE therapy after surgery for primary HCC at EHBH from 2010-2015 were included in Cohort 1. Detailed clinicopathological features of these patients are described in supplementary Table 1. A total of 68 patients received adjuvant sorafenib therapy after surgery for primary HCC at EHBH from 2011-2015 were included in Cohort 2. Detailed clinicopathological features of these patients are described in supplementary Table 2. Another group of 50 HCC specimens were used for analyzing the correlation between SOX1 and miR-361-3p expression. Patient informed consent was obtained and the procedure of human sample collection was approved by the Ethics Committee of EHBH.

### Reverse transcription polymerase chain reaction (RT-PCR)

Total RNA was extracted from the cells using Trizol reagent (Invitrogen, 15596-018). Total cDNAs were synthesized by ThermoScript TM RT-PCR system (Invitrogen, 11146-057). The mRNA amount presented in the cells was measured by semi quantitative RT-PCR. PCR conditions included 1 cycle at 94 °C for 5 minutes, followed by up to 40 cycles of 94 °C for 15 seconds (denaturation), 60 °C for 30 seconds (annealing) and 72 °C for 30 seconds (extension). The sequences of primers used was listed in Supplementary Table 3.

### Luciferase reporter assay

A 300-bp fragment of the *SOX1* 3'UTR containing the conserved miR-361-3p-binding sites was inserted into a luciferase reporter plasmid. The *SOX1* 3'UTR mutant luciferase plasmid contained changes in potential miR-361-3p-binding base sequence “CUGGGGG” to “GACUCUA”. HCC cells were transfected with *SOX1* 3'UTR luciferase reporter in combination with the pRL-TK vector (Promega, E2241) as an internal control. The dual luciferase assay kit was purchased from Promega (0000060417). The luciferase activities were determined using a luminometer (Wallac 1420 Victor 2 multilabel counter system) as described in previous studies 18.

### Western Blotting Assay

The cells collected by cell lysis buffer, then disposed like we described before 19. Equal aliquots of cell extracts were separated on SDS-polyacrylamide gels. The proteins were then transferred to PVDF membranes (Bio-Rad), blocked, and probed with the antibodies. Primary antibody-bound proteins were detected by using an alkaline phosphatase-linked secondary antibody and an ECF Western Blotting system (Amersham). The densitometric analyses of the protein bands vs. the individual loading controls were performed using the ImageQuant 5.2 software (GE Healthcare). The primary antibodies used were listed in supplementary Table 4. The results shown were representative one of three independent experiments.

### Spheroid formation assay

HCC cells were digested and then cultured in a 96-well ultra-low attachment (300 cells per well) and cultured in DMEM/F12 (Gibco) media, supplemented with 1% FBS, 20 ng/mL bFGF and 20 ng/mL EGF for 7 days. The total number of spheres was counted under the microscope (Olympus).

### In vitro limiting dilution assay

Various numbers of Huh7/HepG2 miR-361-3p mimic or miR-361-3p sponge and their control cells were digested and seeded into 96-well ultra-low attachment (2, 4, 8, 16, 32, 64 cells per well, n=8) and cultured in DMEM/F12 (Gibco) supplemented with 1% FBS, 20 ng/mL bFGF and 20 ng/mL EGF for seven days. The CSC proportions were analyzed using Poisson distribution statistics and the L-Calc Version 1.1 software program (Stem Cell Technologies, Inc., Vancouver, Canada) as previously described 20.

### In vivo limiting dilution

For the *in vivo* limiting dilution assay, Huh7 miR-361-3p mimic or Huh7 miR-361-3p sponge and their control HCC cells digested and then mixed with Matrigel (BD) at a ratio of 1:1 and injected subcutaneously at indicated cell doses (1X10^3^, 5X10^3^, 1X10^4^, 5X10^4^) per NOD-SICD mouse (n=6). After two months, tumors formation was evaluated.

### Apoptosis Assay

Huh7 miR-361-3p sponge and control cells were treated with cisplatin (10 μg/ml) or sorafenib (10 μM) for 48 hours, followed by staining with Annexin V and 7-AAD for 15 minutes at room temperature in the dark. Apoptotic cells were determined by an Annexin VFITC Apoptosis Detection Kit I (BD Pharmingen, San Diego, CA) and detected by flow cytometry according to the manufacturer's instructions.

### Statistical Analysis

GraphPad Prism (GraphPad Software, Inc. La Jolla, USA) was used for all statistical analyses. Statistical analysis was carried out using t test or Bonferroni Multiple Comparisons Test: *p<0.05. A p value of less than 0.05 was considered significant.

## Results

### miR-361-3p is upregulated in liver T-ICs

**Figure 1:**
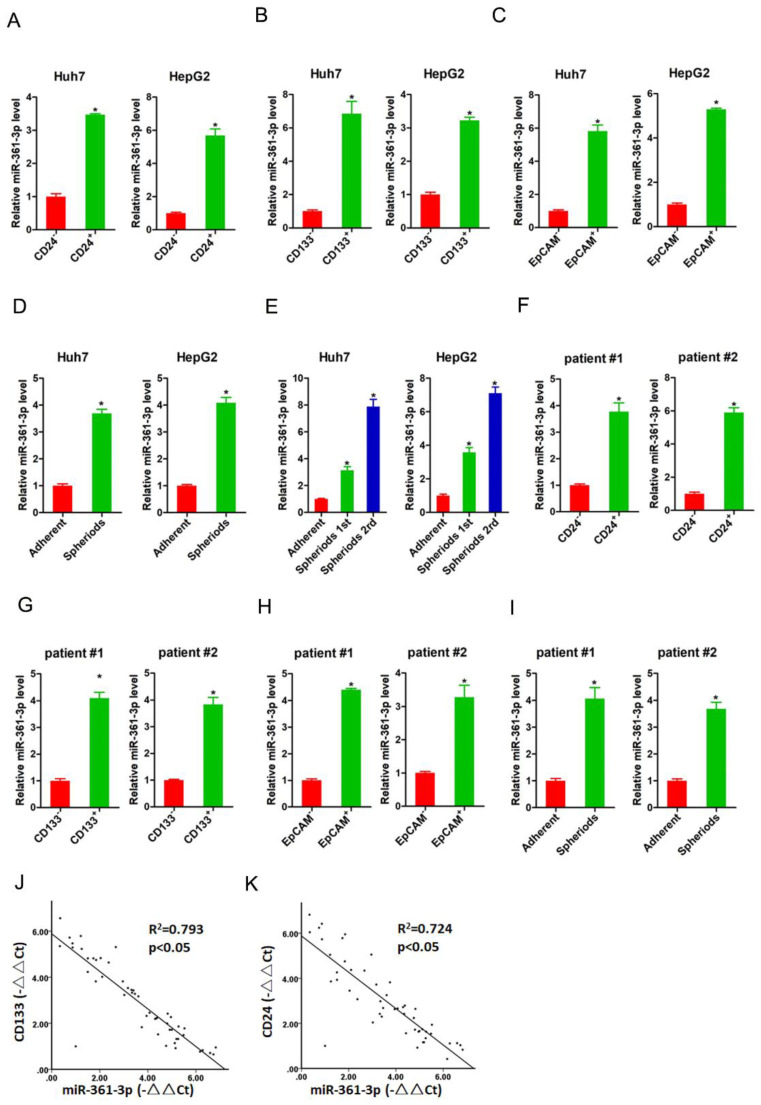
** miR-361-3p was upregulated in liver T-ICs.** A. The expression of miR-361-3p in CD24^+^ and CD24^-^ HCC cells was assessed by real-time PCR assay. B. The expression of miR-361-3p in CD133^+^ and CD133^-^ HCC cells was detected by real-time PCR assay. C. The expression of miR-361-3p in EpCAM^+^ and EpCAM^-^ HCC cells was checked by real-time PCR assay. D. The expression of miR-361-3p in hepatoma spheroids was examined by real-time PCR assay. E. miR-361-3p expression in serial passages of hepatoma spheroids was analyzed by real-time PCR. F. The expression of miR-361-3p in CD24^+^ and CD24^-^ primary HCC cells was assessed by real-time PCR assay. G. The expression of miR-361-3p in CD133^+^ and CD133^-^ primary HCC cells was detected by real-time PCR assay. H. The expression of miR-361-3p in EpCAM^+^ and EpCAM^-^ primary HCC cells was checked by real-time PCR assay. I. The expression of miR-361-3p in primary hepatoma spheroids was examined by real-time PCR assay. J&K. The correlation between the transcription level of miR-361-3p and CD24 or CD133 in fifty HCC tissues was determined by real-time PCR analysis. Data were normalized to U6 or β-actin as △Ct and analyzed by Spearman's correlation analysis.

It was well accepted that CD24, CD133 and EpCAM were liver T-ICs markers 21-23. Therefore, we isolated CD24, CD133 and EpCAM positive HCC cells by flow cytometry. As expected, the expression of miR-361-3p was dramatically increased in CD24, CD133 and EpCAM positive HCC cells compared with their negative HCC cells (Figure 1A-C). Spheroid culture of cancer cells is a routine approach to enrich T-ICs. We observed that expression of miR-361-3p was significantly upregulated in the self-renewing spheroids compared with the attached cells (Figure 1D). In serial passages of Huh7 or HepG2 spheroids, miR-361-3p expression was gradually increased (Figure 1E). Consistently, miR-361-3p expression was also upregulated in CD24, CD133 and EpCAM positive primary HCC cells compared with negative primary HCC cells (Figure 1F-H). The level of miR-361-3p was increased in primary HCC spheroids compared with the attached cells (Figure 1I). More importantly, in HCC tissues, pearson correlation analysis demonstrated that miR-361-3p levels were negatively correlated with the expression of CD24 and CD133 (Figure 1J&K). Taken together, our data demonstrated that miR-361-3p was upregulated in liver T-ICs.

### miR-361-3p promotes liver T-ICs self-renew and tumorigenesis

**Figure 2:**
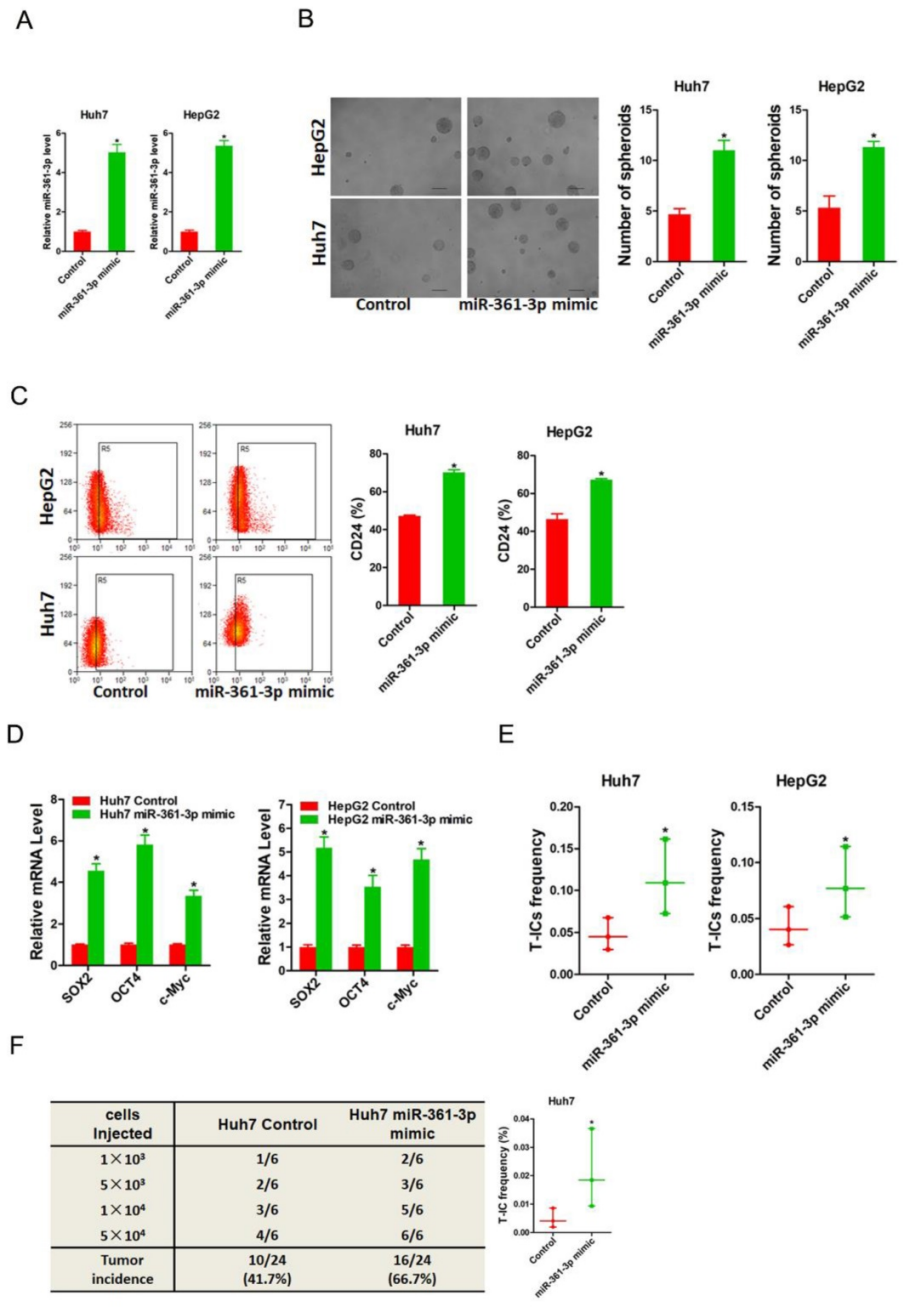
** miR-361-3p promoted liver T-ICs self-renew and tumorigenesis.** A. Huh7 and HepG2 cells were transfected with miR-361-3p mimic virus. The RNA was extracted and identified by real-time PCR. B. Representative images of hepatoma spheroids generated from Huh7/HepG2 miR-361-3p mimic and control cells. The number of spheroids was counted and compared. C. Flow cytometry analysis of CD24^+^ populations in spheroids derived from Huh7/HepG2 miR-361-3p mimic and control cells. Representative results from three independent experiments were shown. D. The expression of transcription factors (SOX2, OCT4 and c-Myc) in Huh7/HepG2 miR-361-3p mimic and control cells were detected by real-time PCR. E. The *in vitro* limiting dilution assay was used to check the population of liver T-ICs in Huh7/HepG2 miR-361-3p mimic and control cells. F. The *in vivo* limiting dilution assay was used to determine the tumorigenicity and liver T-ICs frequency. The frequency of tumor initiating cells was assessed using ELDA software.

To evaluate the potential role of miR-361-3p in liver T-ICs, Huh7 and HepG2 cells were infected with miR-361-3p overexpression virus. The overexpression effect of miR-361-3p was determined by real-time PCR (Figure 2A). Spheroids formation is a routine approach to assess self-renewal ability. We found that Huh7/HepG2 miR-361-3p mimic cells formed much more spheres than control cells (Figure 2B). Moreover, the expression of liver T-ICs markers was upregulated in miR-361-3p mimic HCC cells compared with control cells (Figure 2C). Consistently, the expression of stemness associated transcription factors was increased in miR-361-3p mimic HCC cells compared with control cells (Figure 2D). Furthermore, the *in vitro* and *in vivo* limiting dilution assay indicated that the proportion of liver T-ICs and tumorigenic ability were enhanced in miR-361-3p mimic HCC cells (Figure 2E&F).

### miR-361-3p knockdown inhibits liver T-ICs self-renew and tumorigenesis

**Figure 3:**
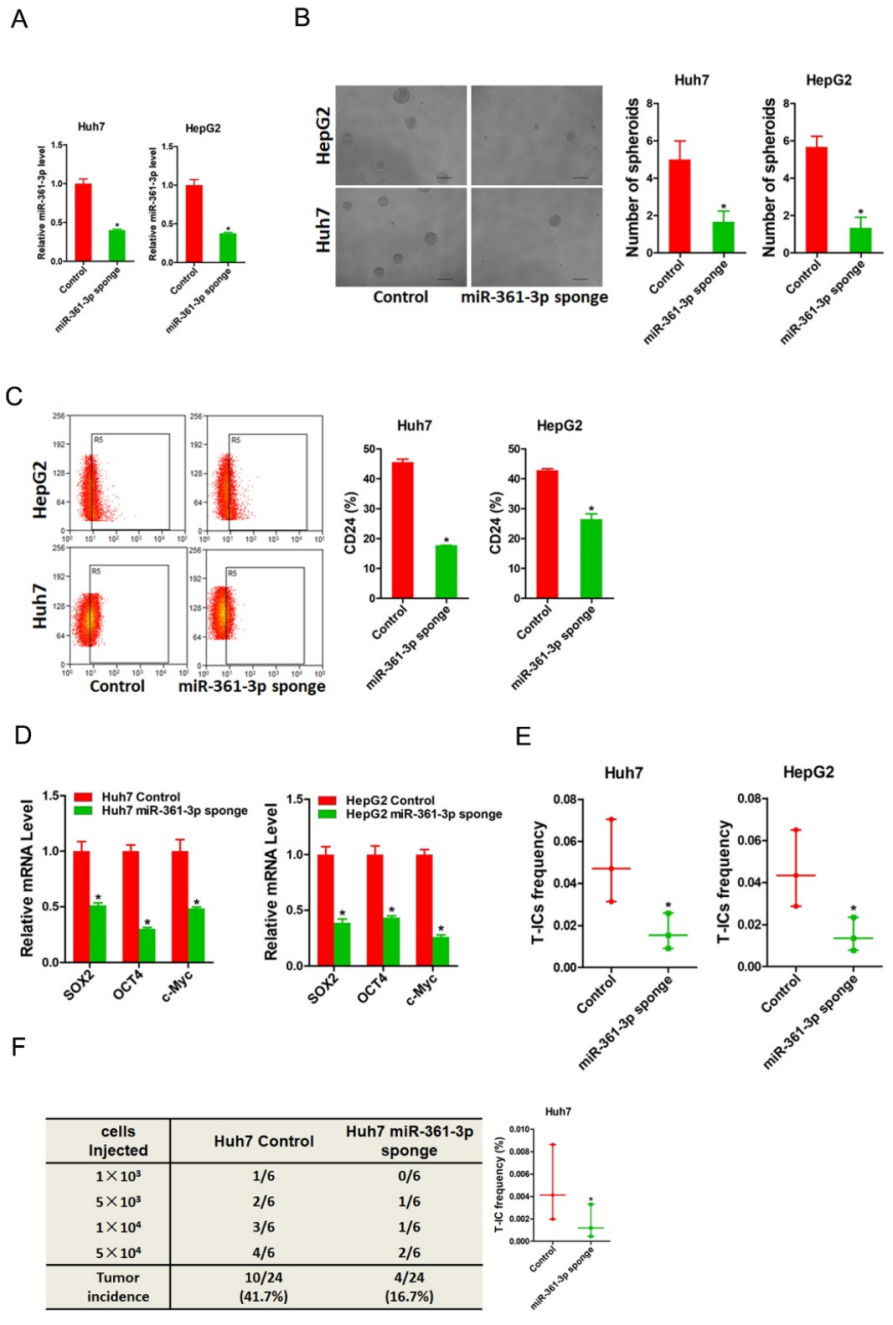
** miR-361-3p knockdown inhibited liver T-ICs self-renew and tumorigenesis.** A. Huh7 and HepG2 cells were transfected with miR-361-3p sponge virus. The RNA was extracted and identified by real-time PCR. B. Representative images of hepatoma spheroids generated from Huh7/HepG2 miR-361-3p sponge and control cells. The number of spheroids was counted and compared. C. Flow cytometry analysis of CD24^+^ populations in spheroids derived from Huh7/HepG2 miR-361-3p sponge and control cells. Representative results from three independent experiments were shown. D. The expression of transcription factors (SOX2, OCT4 and c-Myc) in Huh7/HepG2 miR-361-3p sponge and control cells were detected by real-time PCR. E. The *in vitro* limiting dilution assay was used to check the population of liver T-ICs in Huh7/HepG2 miR-361-3p sponge and control cells. F. The *in vivo* limiting dilution assay was used to determine the tumorigenicity and liver T-ICs frequency. The frequency of tumor initiating cells was assessed using ELDA software.

To further explore the role of miR-361-3p in liver T-ICs, Huh7 and HepG2 cells were infected with miR-361-3p interference virus. The knockdown effect of miR-361-3p was determined by real-time PCR (Figure 3A). Spheroids formation is a routine approach to assess self-renewal ability. We found that Huh7/HepG2 miR-361-3p knockdown cells formed much less spheres than control cells (Figure 3B). Moreover, the expression of liver T-ICs markers was downregulated in miR-361-3p knockdown HCC cells compared with control cells (Figure 3C). Consistently, the expression of stemness associated transcription factors was decreased in miR-361-3p knockdown HCC cells compared with control cells (Figure 3D). Furthermore, the *in vitro* and *in vivo* limiting dilution assay indicated that the proportion of liver T-ICs and tumorigenic ability were impaired in miR-361-3p knockdown HCC cells (Figure 3E&F). Collectively, our results demonstrated that miR-361-3p promoted liver T-ICs expansion.

### SOX1 is required in miR-361-3p-mediated liver T-ICs expansion

**Figure 4:**
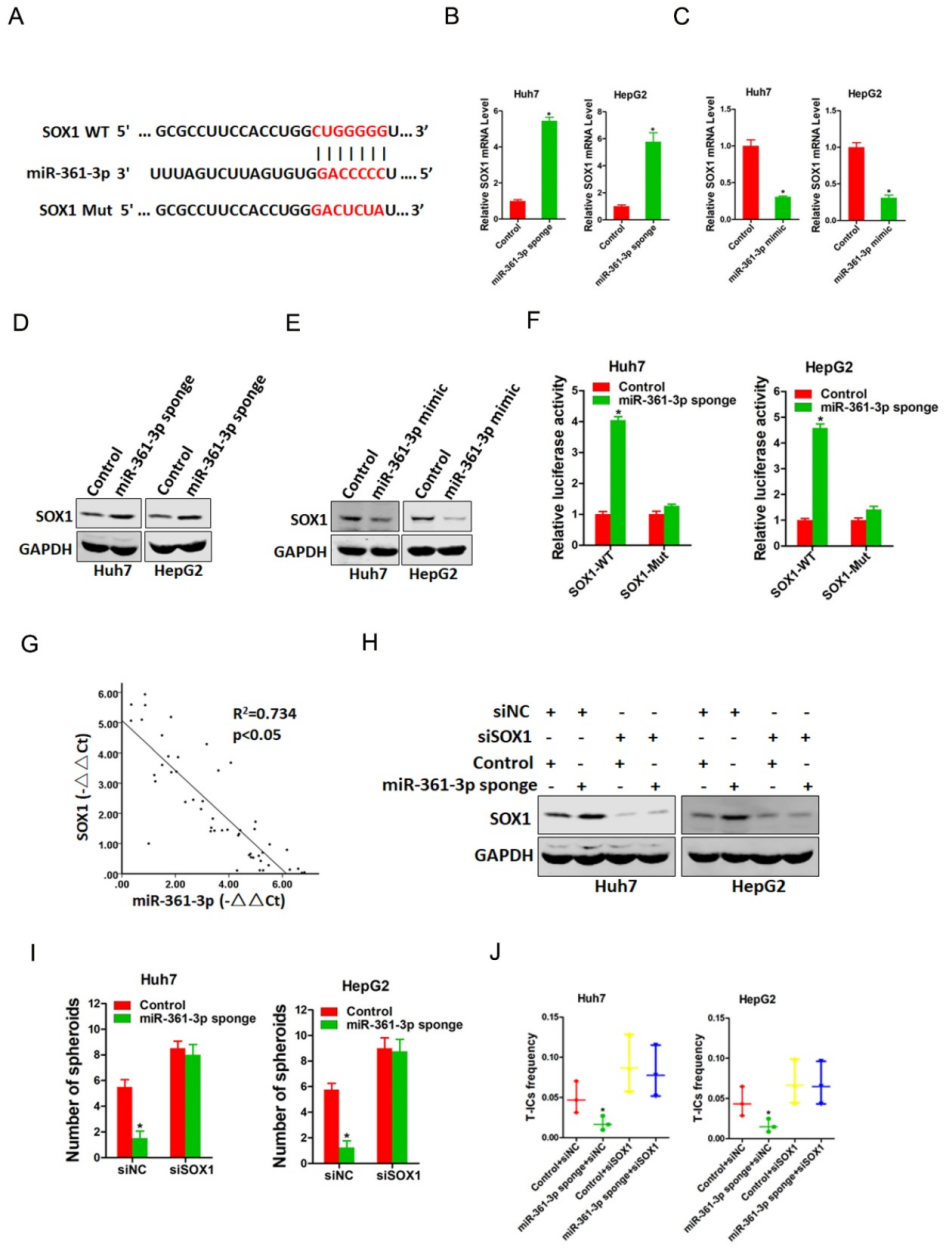
** miR-361-3p directly targeted SOX1 in liver T-ICs.** A. Schematic diagram of luciferase plasmids of SOX1 3'-UTR wild-type (WT) and SXO1 3'-UTR mutant (MUT). B. The SOX1 mRNA expression in Huh7/HepG2 miR-361-3p sponge and control cells were detected by real-time PCR. C. The SOX1 mRNA expression in Huh7/HepG2 miR-361-3p mimic and control cells were detected by real-time PCR. D. The SOX1 protein expression in Huh7/HepG2 miR-361-3p sponge and control cells were detected by western blot. E. The SOX1 protein expression in Huh7/HepG2 miR-361-3p mimic and control cells were detected by western blot. F. HCC cells were stably transfected with SOX1 3'-UTR wild-type (WT) and SXO1 3'-UTR mutant (MUT) luciferase reporter for 24 hours. The luciferase activity was measured as described in “materials and methods”. G. A negative correlation was observed between miR-361-3p and SOX1 expression in 50 clinical samples of HCC (R^2^ = 0.734, P < 0.05, by Spearman's correlation analysis). H. Huh7/HepG2 miR-361-3p sponge and control cells were transfected with siRNA SOX1 or its vector control. The stable transfectants were identified by Western Blotting. I. Huh7/HepG2 miR-361-3p sponge and control cells transfected with shRNA SOX1 and then subjected to spheroids formation assay. J. Huh7/HepG2 miR-361-3p sponge and control cells transfected with shRNA SOX1 and then subjected to *in vitro* limiting dilution assay.

To elucidate mechanism underlying miR-361-3p-mediated liver T-ICs expansion, TargetScan and miRbase were used to predicted the potential targets of miR-361-3p in liver T-ICs. Bioinformatics analysis found that miR-361-3p has a putative binding site in SOX1 mRNA 3'-UTR (Figure 4A). As expected, both the mRNA and protein expression of SOX1 were upregulated in miR-361-3p knockdown HCC cells and downregulated in miR-361-3p overexpression HCC cells (Figure 4B-E). Luciferase reporter assays with a vector that included the wild-type (WT) 3'-UTR or mutant-type (MUT) 3'-UTR of SOX1 were then performed to determine whether miR-361-3p could directly regulate SOX1. The results showed that miR-361-3p knockdown significantly enhanced the relative luciferase activity compared with control group. However, such effects were diminished when the predicted binding site was mutated (Figure 4F). These results indicated that miR-361-3p directly suppressed SOX1 expression by binding to its 3'-UTR. In addition, a significant negative correlation was identified between SOX1 and miR-361-3p expression in clinical samples of HCC (R^2^ = 0.734, P < 0.05, n=50) (Figure 4G).

Next, we explore the expression of SOX1 in liver T-ICs. As shown in supplementary Figure 1A-C, SOX1 expression was reduced in CD24^+^, CD133^+^ and EpCAM^+^ liver T-ICs that were sorted from HCC cell lines. Compared with the attached cells, SOX1 expression was downregulated in HCC spheres derived from HCC cells (supplementary Figure 1D). Moreover, SOX1 expression was decreased in CD24^+^, CD133^+^ and EpCAM^+^ liver T-ICs that were sorted from primary HCC patients (supplementary Figure 1E-G). Consistently, we also found that SOX1 expression was downregulated in HCC spheres derived from primary HCC patients (supplementary Figure 1H). To further confirm whether SOX1 was required for miR-361-3p mediated liver T-ICs expansion, the special SOX1 siRNA was used (Figure 4H). SOX1 siRNA abrogated the self-renewal ability and liver T-ICs frequency between miR-361-3p knockdown HCC cells and control cells (Figure 4I&J).

### miR-361-3p knockdown HCC cells are sensitive to cisplatin and sorafenib treatment

**Figure 5:**
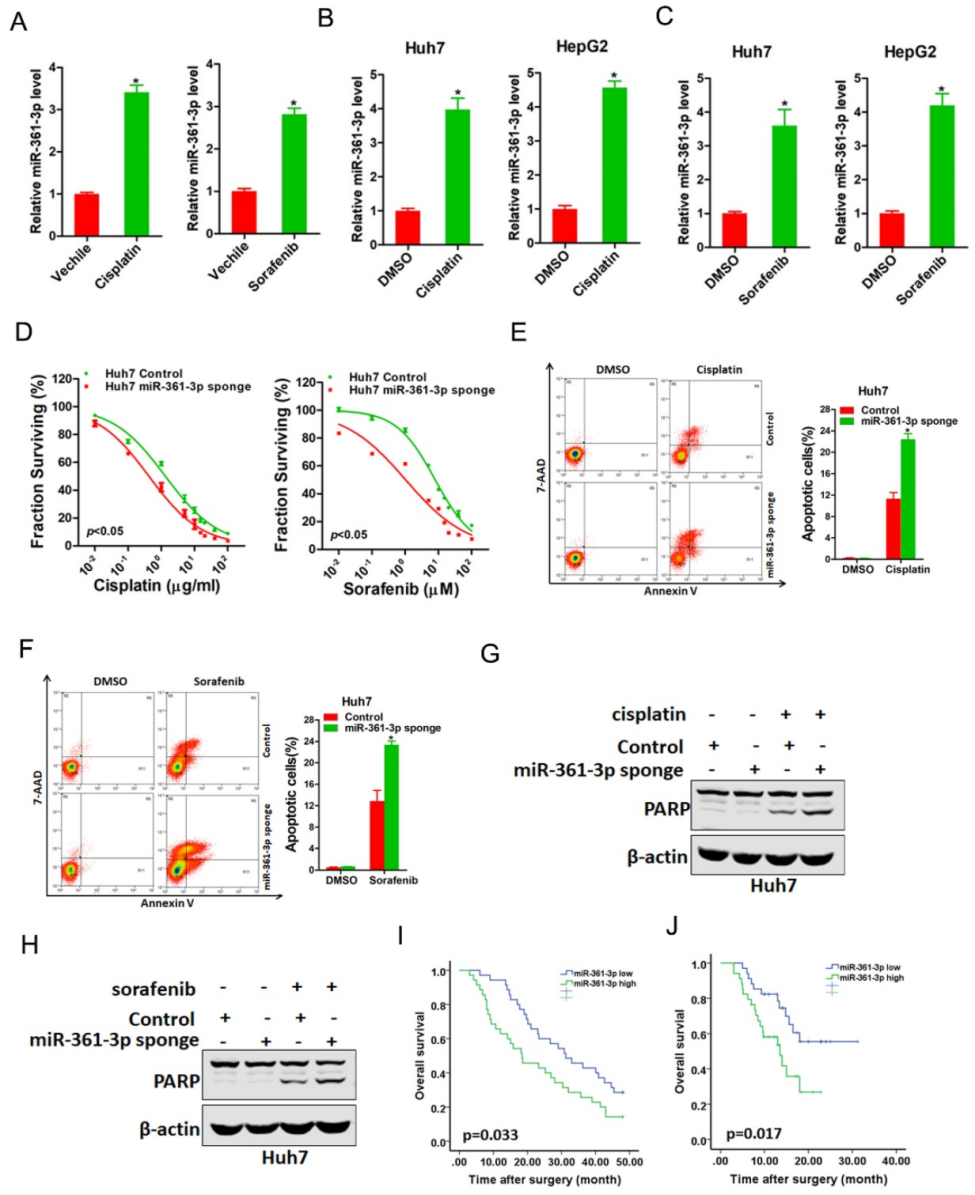
** miR-361-3p knockdown HCC cells were sensitive to cisplatin and sorafenib treatment.** A. The expression of miR-361-3p in cisplatin-resistant or sorafenib-resistant xenograft was measured by real-time PCR. B. The expression of miR-361-3p in cisplatin-resistant HCC cell lines was measured by real-time PCR. C. The expression of miR-361-3p in sorafenib-resistant HCC cell lines was measured by real-time PCR. D. Cell survival curves of Huh7 miR-361-3p sponge and control cells treated with cisplatin or sorafenib. E. Huh7 miR-361-3p sponge and control cells were treated with cisplatin (4 μg/ml) for 48 hours. The apoptotic cells were detected by flow cytometry. F. Huh7 miR-361-3p sponge and control cells were treated with sorafenib (10 μM) for 48 hours. The apoptotic cells were detected by flow cytometry. G. Huh7 miR-361-3p sponge and control cells were treated with cisplatin (4 μg/ml) for 48 hours. The cell extracts were subjected to western blotting with special antibody against PARP. H. Huh7 miR-361-3p sponge and control cells were treated with sorafenib (10 μM) for 48 hours. The cell extracts were subjected to western blotting with special antibody against PARP. I. The total of 70 HCC patients (Cohort 1) were divided into low miR-361-3p group (n = 35) and high miR-361-3p group (n = 35), and the overall survival of the patients in the two groups were compared. J. The total of 68 HCC patients (Cohort 2) were divided into low miR-361-3p group (n = 34) and high miR-361-3p group (n = 34), and the overall survival of the patients in the two groups were compared.

We next explored whether miR-361-3p was involved in the regulation of chemo-resistance of HCC. As expected, miR-361-3p expression was markedly upregulated in cisplatin-resistance or sorafenib-resistance xenograft (Figure 5A). Consistently, miR-361-3p expression was also significantly increased in cisplatin-resistance or sorafenib-resistance HCC cell lines (Figure 5B&C). Moreover, the sensitivity of cisplatin or sorafenib was increased in miR-361-3p knockdown HCC cells compared with control HCC cells (Figure 5D). We also observed that interference of miR-361-3p sensitized HCC cells to undergo cisplatin-induced or sorafenib-induced cell apoptosis (Figure 5E-H). Furthermore, Kaplan-Meier analysis revealed the survival benefits in adjuvant TACE-treated or sorafenib-treated HCC patients with low miR-361-3p levels (Figure 5I&J).

## Discussion

Hepatocellular carcinoma (HCC) is the sixth most common cancer in the world, accounting for 90% of human liver cancer 24. The incidence of liver cancer is rising due to various factors such as hepatitis, alcoholic fatty liver, nonalcoholic fatty liver and aflatoxin 25, 26. Hepatectomy and liver transplantation are commonly used in patients with early stage HCC 27. However, most patients with advanced HCC are contradicting for surgery, and the survival benefit of TACE or targeted drug sorafenib is limited 28. Therefore, it is necessary to further clarify the development of HCC. In this study, we confirmed for the first time that miR-361-3p was highly expressed in liver T-ICs, which enhanced liver T-ICs self-renewal and tumorigenesis ability. Our clinical data showed that miR-361-3p could be used to predict TACE and sorafenib response in HCC patients.

MiRNAs, a small non-coding RNA molecule (containing about 22 nucleotides), regulates RNA silencing and post-transcriptional of gene expression 29. miRNAs act as oncogenes or tumor suppressors in tumors dependent on special conditions. miR-361-3p has been reported upregulated in various human tumors, including breast cancer, oral squamous cancer and pancreatic cancer 16, 17, 30. However, miR-361-3p functions in liver T-ICs have never been investigated before. In current studies, we found that miR-361-3p was upregulated in liver T-ICs. Moreover, we found that miR-361-3p knockdown inhibited liver T-ICs self-renew and tumorigenesis. Conversely, forced miR-361-3p expression promoted liver T-ICs self-renew and tumorigenesis.

SOX1, belonging to SRY (sex determining region Y)-box (SOX) family proteins, is evolutionarily conserved in many species and participated as a key regulator of neural cell fate determination and differentiation 31, 32. Previous studies have demonstrated that SOX1 plays an essential role in liver cancer progression 33. In addition, SOX1 was also reported to be involved in the regulation of cancer stemness 34. We hereby found that miR-361-3p knockdown increased SOX1 mRNA and protein expression in liver T-ICs. Moreover, we found that miR-361-3p directly regulates SOX1 expression via interaction with its 3' UTR. In addition, a significant negative correlation was identified between SOX1 and miR-361-3p expression in clinical samples of HCC. SOX1 siRNA could diminish the self-renewal ability and liver T-ICs frequency between miR-361-3p knockdown HCC cells and control cells.

For advanced HCC patients, TACE (transcatheter arterial chemoembolization) and the targeted drug sorafenib are the major means of treatment for a long time. However, only a small proportion of patients respond to sorafenib or TACE, and the majority of patients not only have no curative effect, but also have serious side effects 35, 36. Therefore, elucidating the mechanism of TACE and sorafenib resistance has become an important link to prolong the survival time of HCC patients. Due to the different responses to sorafenib and TACE in different HCC patients, how to select biomarkers to predict drug reactivity has become a key issue to improve the clinical efficacy of sorafenib and TACE. In the present study, our finding revealed that miR-361-3p knockdown of HCC cells is more sensitive to cisplatin or sorafenib treatment. The TACE or sorafenib cohort analysis further indicated that a low miR-361-3p level in HCC patients can serve as a reliable predictor for TACE or sorafenib response.

In conclusion, we demonstrate that miR-361-3p is upregulated in liver T-ICs, which in turn promotes the self-renewal and tumorigenicity of liver T-ICs. In addition, miR-361-3p promotes liver T-ICs expansion through directly regulating SOX-1. Moreover, miR-361-3p knockdown HCC cells are more sensitive to cisplatin or sorafenib treatment. These data provide insight into the miR-361-3p as a potential therapeutic target against liver T-ICs and a potential predictor for TACE or sorafenib treatment of HCC patients.

## Supplementary Material

Supplementary figure and tables.

## Abbreviations

T-ICs: tumor-initiating cells
CSCs: cancer stem cells
miRNAs: MicroRNAs
3'-UTR: 3'-untranslational region
DMEM: Dulbecco's modified Eagle's medium
FBS: fetal bovine serum
APC: adenomatous polyposis coli
TACE: transcatheter arterial chemoembolization
HCC: hepatocellular carcinoma
EHBH: Eastern Hepatobiliary Surgery Hospital
RT-PCR: Reverse transcription polymerase chain reaction
SOX: SRY (sex determining region Y)-box
EpCAM: epithelial cell adhesion molecule
CD24: cluster of differentiation 24
CD133: cluster of differentiation 133
