# Supplementary material for: miR-361-3p Regulates Liver Tumor-initiating Cells Expansion and Chemo-resistance
Source: J Cancer. 2021 Jan 1;12(5):1483–92. doi: 10.7150/jca.52395 (PMC7847642; doi:10.7150/jca.52395)
Supplement: Supplementary file 1 — Supplementary figure and tables. [file jcav12p1483s1.pdf]

miR-361-3p Regulates Liver Tumor-initiating Cells Expansion and Chemo-resistance

Shuping Qu[1,#], Xiaobing Zhang[1,#], Yue Wu[1, #], HengYu Li[2], Jian Zhai[1,*], Dong Wu[1,*]

[1]Department of Hepatic Surgery, Third Affiliated Hospital of Second Military Medical University, Shanghai, 200438, China.
[2]Department of General surgery, First Affiliated Hospital of Second Military Medical University, Shanghai, 200433, China.

[#]These authors contributed equally to this work.

Correspondence author address: Department of Hepatic Surgery, Third Affiliated Hospital of Second Military Medical University, Shanghai, 200438, China, E-mail: jianzhai1979@126.com (Jian Zhai); WuDongEHBH@126.com (Dong Wu).

Running title: miR-361-3p promotes liver T-ICs expansion.

Funding

This work is supported by the National Natural Science Foundation of China (81672718).

Supplementary Figure 1

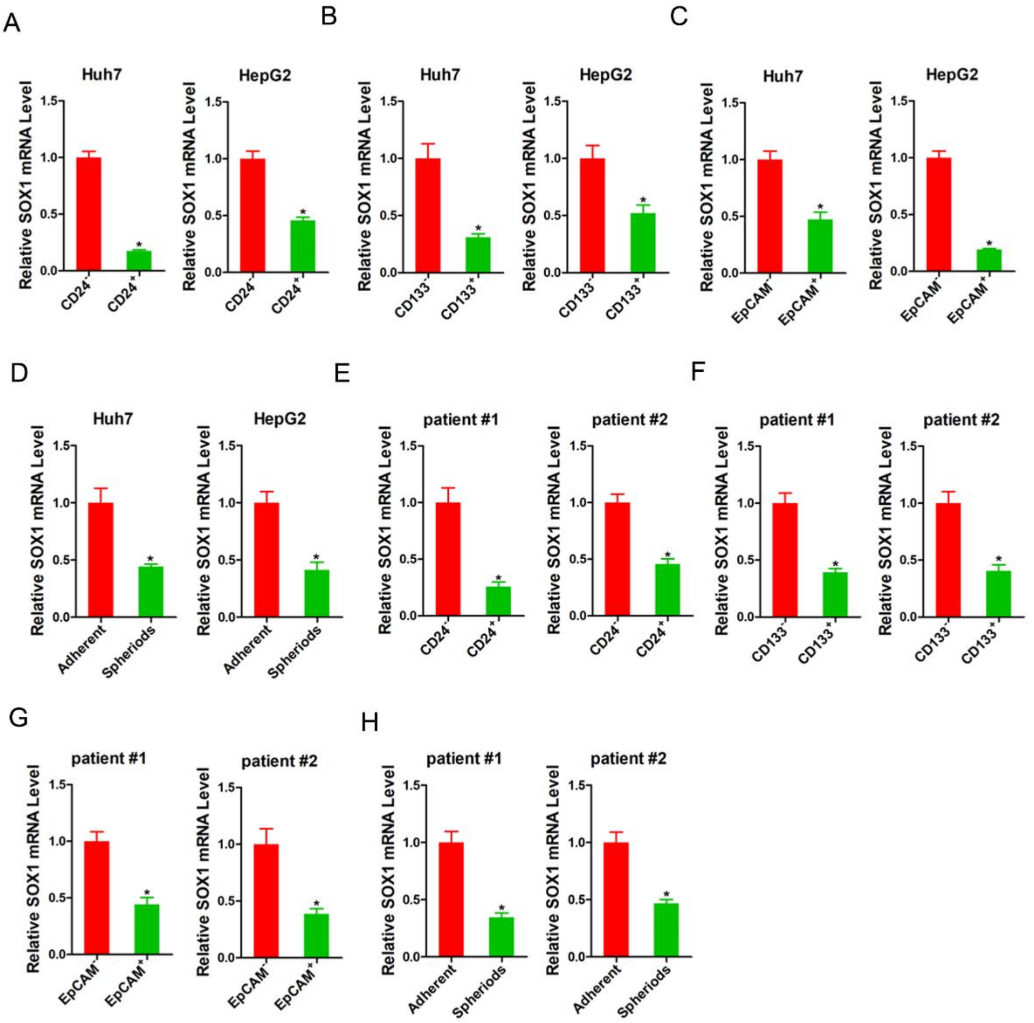

Supplementary Figure 1:

A. The expression of SOX1 in CD24^{+} and CD24^{-} HCC cells was assessed by real-time PCR assay.

B. The expression of SOX1 in CD133^{+} and CD133^{-} HCC cells was detected by real-time PCR assay.

C. The expression of SOX1 in EpCAM^{+} and EpCAM^{-} HCC cells was checked by real-time PCR assay.

D. The expression of SOX1 in hepatoma spheroids was examined by real-time PCR assay.

E. The expression of SOX1 in CD24^{+} and CD24^{-} primary HCC cells was assessed by

real-time PCR assay.

F. The expression of SOX1 in $CD133^+$ and $CD133^-$ primary HCC cells was detected by real-time PCR assay.

G. The expression of SOX1 in $EpCAM^+$ and $EpCAM^-$ primary HCC cells was checked by real-time PCR assay.

H. The expression of SOX1 in primary hepatoma spheroids was examined by real-time PCR assay.

Supplementary Tables

Supplementary Table 1. Clinicopathological features of 70 HCC patients in Cohort 1

Characteristics		Total n=70
Age(year)	≤50	44
	>50	26
Gender	Male	65
	Female	5
HBsAg	Positive	57
	Negative	13
AFP(μg/L)	≤400	51
	>400	19
Tumor size(cm)	≤5	26
	>5	44
Tumor number	Single	43
	Multiple	27
Portal vein tumor thrombus	Yes	12
	No	58
Pathological satellite	Yes	28
	No	42
BCLC stage	A	29
	B or C	41
TNM	I-II	30
	I-II	40

HBsAg: hepatitis B virus surface antigen; AFP: α-fetoprotein; TNM: Tumor-Nodes-Metastasis; BCLC: Barcelona Clinic Liver Cancer Staging.

Supplementary Table 2. Clinicopathologic Features of 68 HCC Specimens in Cohort 2

Characteristics		Total n=68
Age(year)	≤50	40
	>50	28
Gender	Male	61
	Female	7
HBsAg	Positive	57
	Negative	11
AFP(μg/L)	≤400	50
	>400	18
Tumor size(cm)	≤5	19
	>5	49
Tumor number	Single	38
	Multiple	30
Portal vein tumor thrombus	Yes	10
	No	58
Pathological satellite	Yes	25
	No	43
BCLC stage	A	26
	B or C	42
TNM	I-II	29
	I-II	39

HBsAg: hepatitis B virus surface antigen; AFP: α-fetoprotein; TNM: Tumor-Nodes-Metastasis; BCLC: Barcelona Clinic Liver Cancer Staging.

Supplementary Table 3. Primer List.

Gene	Forward primer	Reverse primer (5'-3')
miR-361-3p	Forward	GCCGCTCCCCCAGGTGTGATT
	Reverse	GTGCAGGGTCCGAGGT
U6	Forward	CTCGCTTCGGCAGCACATA
	Reverse	AACGATTCACGAATTTGCGT
β-actin	Forward	GGCCCAGAATGCAGTTCGCCTT
	Reverse	AATGGCACCCTGCTCACGCA
SOX2	Forward	TGGAGAAGGAATGGTCCACTTC
	Reverse	GGATAAGTACACGCTGCCCG
OCT4	Forward	ATGTGCGCGTAACTGTCCAT
	Reverse	CTGCAGTGTGGGTTTCGGGCA
SOX1	Forward	AATACTGGAGACGAACGCCG
	Reverse	CCCTCGAGCAAAGAAAACGC
c-Myc	Forward	CCCTCCACTCGGAAGGACTA
	Reverse	GCTGGTGCATTTTCGGTTGT

Supplementary Table 4. Antibody List.

Antigens	Manufacturer	Application
SOX1	Proteintech Group, China	1:1000 for WB
PARP	Cell Signaling Technology, Beverly, MA	1:1000 for WB
β-actin	Santa Cruz Biotechnology, CA	1:5000 for WB
GAPDH	Santa Cruz Biotechnology, CA	1:5000 for WB
